# Feasibility of Dose Escalation in Patients With Intracranial Pediatric Ependymoma

**DOI:** 10.3389/fonc.2019.00531

**Published:** 2019-06-21

**Authors:** Fatima Tensaouti, Anne Ducassou, Léonor Chaltiel, Stéphanie Bolle, Jean Louis Habrand, Claire Alapetite, Bernard Coche-Dequeant, Valérie Bernier, Line Claude, Christian Carrie, Laetitia Padovani, Xavier Muracciole, Stéphane Supiot, Aymeri Huchet, Julie Leseur, Christine Kerr, Grégorie Hangard, Albert Lisbona, Farid Goudjil, Régis Ferrand, Anne Laprie

**Affiliations:** ^1^ToNIC, Toulouse NeuroImaging Center, Universite de Toulouse, Inserm, Toulouse, France; ^2^Department of Radiation Oncology, Institut Claudius Regaud, Institut Universitaire du, Cancer de Toulouse-Oncopole, Toulouse, France; ^3^Department of Biostatistics, Institut Claudius Regaud, Institut Universitaire du Cancer de Toulouse-Oncopole, Toulouse, France; ^4^Department of Radiotherapy Oncology, Institut Gustave Roussy, Villejuif, France; ^5^Department of Radiation Oncology, Centre Francois Baclesse, Caen, France; ^6^Department of Radiation Oncology, Institut Curie, Paris, France; ^7^Department of Radiation Oncology, Oscar Lambret Centre, Lille, France; ^8^Department of Radiation Oncology, Centre Alexis Vautrin, Vandœuvre-lès-Nancy, France; ^9^Department of Radiation Oncology, Centre Léon Bérard, Lyon, France; ^10^Department of Radiation Oncology, CHU La Timone, Marseille, France; ^11^Department of Radiation Oncology, Institut de Cancerologie de l'Ouest, Nantes, France; ^12^Department of Radiation Oncology, Centre Hospitalier et Universitaire, Bordeaux, France; ^13^Department of Radiation Oncology, Centre Eugéne Marquis, Rennes, France; ^14^Department of Radiation Oncology, Institut Regional du Cancer Montpellier, Val d'Aurelle, Montpellier, France; ^15^Department of Engineering and Medical Physics, Institut Universitaire du Cancer de Toulouse-Oncopole, Toulouse, France; ^16^Université Toulouse III Paul Sabatier, Toulouse, France

**Keywords:** photon therapy, proton therapy, boost, treatment planning, ependymoma, intracranial

## Abstract

**Background and purpose:** Pediatric ependymoma carries a dismal prognosis, mainly owing to local relapse within RT fields. The current prospective European approach is to increase the radiation dose with a sequential hypofractionated stereotactic boost. In this study, we assessed the possibility of using a simultaneous integrated boost (SIB), comparing VMAT vs. IMPT dose delivery.

**Material and methods:** The cohort included 101 patients. The dose to planning target volume (PTV59.4) was 59.4/1.8 Gy, and the dose to SIB volume (PTV67.6) was 67.6/2.05 Gy. Gross tumor volume (GTV) was defined as the tumor bed plus residual tumor, clinical target volume (CTV59.4) was GTV + 5 mm, and PTV59.4 was CTV59.4 + 3 mm. PTV67.6 was GTV+ 3 mm. After treatment plan optimization, quality indices and doses to target volume and organs at risk (OARs) were extracted and compared with the standard radiation doses that were actually delivered (median = 59.4 Gy [50.4 59.4]).

**Results:** In most cases, the proton treatment resulted in higher quality indices (*p* < 0.001). Compared with the doses that were initially delivered, mean, and maximum doses to some OARs were no higher with SIB VMAT, and significantly lower with protons (*p* < 0.001). In the case of posterior fossa tumor, there was a lower dose to the brainstem with protons, in terms of V59 Gy, mean, and near-maximum (D2%) doses.

**Conclusion:** Dose escalation with intensity-modulated proton or photon SIB is feasible in some patients. This approach could be considered for children with unresectable residue or post-operative FLAIR abnormalities, particularly if they have supratentorial tumors. It should not be considered for infratentorial tumors encasing the brainstem or extending to the medulla.

## Introduction

Pediatric ependymoma is a frequent brain tumor in children, adolescents, and young adults. Treatment relies on maximum surgical resection, followed by localized radiotherapy at 59.4/1.8 Gy (1–3).

Intensity-modulated photon radiotherapy (IMRT) is currently used to reduce the dose to organs at risk (OARs). However, on account of its physical properties, proton-beam therapy (PBT) produces excellent dose distribution and often decreases the dose to OARs, potentially lowering the risk of radiation-induced secondary cancers for those pediatric patients who live well-beyond the end of their treatment (4–6).

Several studies have reported the advantages of PBT over different forms of photon radiotherapy, including IMRT (7–10). MacDonald et al. found that proton beams and IMRT had similar target coverage, but normal tissue sparing was better with IMPT for patients with ependymoma (11, 12). Pulsifer et al. (13) reported that early cognitive outcomes following PBT for pediatric central nervous system (CNS) tumors were encouraging, compared with published outcomes for photon radiotherapy.

However, despite the aggressiveness of the treatment and improvements in radiation techniques for this tumor, outcomes remain poor, with 5 year event-free survival and overall survival rates of 50 and 75% (14). Chemotherapy does not seem to be effective for this tumor. It is only used in very young patients to avoid irradiation and in children with residual disease after initial resection (15), although it is currently being assessed in the SIOP trial as a maintenance treatment. Based on a large national cohort study (16, 17), our team and others found that the majority of relapses occur within the high-dose regions (10, 16). Moreover, a survival benefit of stereotactic radiotherapy (SRT) to residue was described in a prospective clinical trial (18). This prompted us to examine a new dose escalation approach, with a view to sparing the surrounding OARs-particularly the brainstem. We therefore, undertook an *in-silico* dosimetric comparison between volumetric modulated arc therapy (VMAT) photon therapy and intensity-modulated proton therapy (IMPT), in order to identify the types of patients who would benefit from this escalation.

## Materials and Methods

### Patients

The cohort included 101 patients drawn from the PEPPI national multicenter database. These patients were treated in France's 13 national pediatric radiotherapy reference centers between 2000 and 2013. We were able to retrospectively replan 91 of them with VMAT or IMPT. Those patients who were not replanned (10 patients) were not eligible for dose escalation, as their brainstem would have been exposed to radiation on account of their tumor size or location (see Supplementary Figure 1 for three examples). This study was approved by the national French ethics committee and the French data protection authority (CNIL: Commission nationale de l'informatique et des libertés de France).

### Target Volumes and Organs at Risk

The gross tumor volume (GTV) was kept as originally delineated, defined as the tumor bed (border of the surgical cavity, tissues, and anatomical areas initially involved with disease) plus residual disease when present. Clinical target volume for 59.4 Gy (relative biological effectiveness, RBE) (CTV_59.4_) was GTV + 5 mm, and planning target volume (PTV_59.4_) was CTV_59.4_ + 3 mm. The PTV of 67.6 Gy (cobalt gray equivalent, CGE) (PTV_67.6_) was GTV+ 3 mm.

The dose to PTV_59.4_ was 59.4/1.8 Gy, and the dose to simultaneous integrated boost (SIB) volume (PTV_67.6_) was 67.6/2.05 Gy (equivalent to sequential boost in clinical trial NCT 02265770). We used a constant RBE value of 1.10 for prescribed doses. It should be noted that most of the other absorbed doses described here were relative doses, normalized to the prescribed dose.

As the data we collected came from patients attending 13 different pediatric radiation oncology reference centers, OAR delineation varied. To be able to compare dose distributions, an experienced radiation oncologist (AL) retrospectively added and corrected OARs so that all patients had the same set. The following 11 OARs were defined: brain, hippocampus, brainstem, inner ears, optic nerves, chiasm, eyes and lenses, pituitary gland, spinal cord, cerebellum, and temporal lobes.

### Treatment Planning

Photon and proton dose plans were generated for all patients in a research version of the RayStation v5 treatment planning system (RaySearch Laboratories, Stockholm, Sweden). The CT calibration curves specific to each center were retrieved before dose calculation.

The photon plans were generated as dual-arc 6 MV VMAT plans, using beam data from a linear accelerator (Varian, Palo Alto, CA, USA). The photon dose was calculated with the clinically used collapsed cone algorithm. The proton plans were pencil-beam scanning (PBS) plans with two to three beams, using beam data obtained from a PBS-dedicated nozzle (Ion Beam Applications, Louvain-la-Neuve, Belgium). A range shifter (water equivalent thickness: 74.1 mm) was used for all the beams in all the treatment plans, as the skin-to-target distance was always <7 cm. All proton plans were optimized with IMPT (multifield optimization). The physical proton dose was optimized. For all photon and proton plans, the prescribed dose was set at the D50% of the PTV_67.6_. The aim for the treatment plans was to achieve a dose distribution comparable to photons or better. All treatment plans were optimized with the physical dose-volume objectives/constraints (for targets as well as OARs). The PTV coverage goals and clinical goals for the OARs are provided in Supplementary Tables 1, 2. All proton and photon plans were created by the same physicist and validated by the same radiation oncologist.

### Evaluation

We analyzed three dose-volume descriptors for PTVs: D98, D50, and D2%.

Mean doses and relevant volume-dose values were derived for each OAR.

In addition, several International Commission on Radiation Units and Measurements (ICRU) indices (19), including homogeneity (HI), coverage (CO), conformity (CI), target coverage (TCO), and dice similarity coefficient (DSC), were derived from the dose-volume histograms.

Treated volume (V95% for the body), and integral dose (mean dose for the body) were calculated for each treatment plan (20, 21). All these data were extracted using a script developed with the RaySearch Laboratories team.

Additionally, we compared the mean and near-maximum doses to eight OARs (brainstem, pituitary gland, hippocampus, temporal lobes, chiasm, inner ears, eyes, optic nerves) calculated *in silico* for SIB dose escalation using VMAT and IMPT with those that were actually delivered to the patients in this cohort, including all the prescribed doses (Table 1) and all the radiation techniques (3D, IMRT, VMAT, IMRT, tomotherapy, protons, and mixed photon-proton).

**Table 1:** Patient and tumor characteristics.

**Patients (*N* = 91)**	**Infratentorial**	**Supratentorial**
Number	60 (65.93%)	31 (34.07%)
**Extent of resection**		
GTR	51 (85.0%)	28 (90.3%)
STR	9 (15.0%)	3 (9.7%)
**Prescribed doses at treatment (Gy)**		
Median (range)	59.4 (50.4-66)	59.4 (54-60)
Doses <59.4 Gy	27 (41.67%)	10 (32.3%)
Doses ≥ 59.4 Gy	33 (55.0 %)	21 (67.7%)
**Radiation techniques at treatment**		
CRT-3D	25 (41.67%)	15 (48.39%)
IMRT	28 (46.67%)	11 (35.48 %)
Proton	5 (8.33%)	5 (16.13%)
Mixed proton-photon	2 (3.33%)	0 (0%)
**PTV**_**67.6Gy**_ **(CC)**		
Median volume (range)	18.43 (2.61-121.35)	39.12 (15.26-156.59)
**PTV**_**59.4Gy**_ **(CC)**		
Median volume (range)	48.90 (10.19-224.10)	81.1 (37.27-253.81)

### Statistics

Data were summarized by frequency and percentage for categorical variables, and by median and range for continuous variables. Comparisons between the two treatment plan modalities were performed using the Wilcoxon signed-rank test for paired data. The Benjamini-Hochberg correction was applied for multiple comparisons. For each comparison, we computed the difference between IMPT and VMAT for each patient (Δ). All reported *p*-values were two-sided. For all statistical tests, differences were considered significant at the 5% level. Statistical analyses were performed using STATA 13.1 and R 3.5.1 software.

## Results

### Patients Excluded From the Study

We excluded 10 patients from the present dose escalation study because the location of their tumor meant that a substantial volume of brainstem was concerned. Four of them had a large tumor in close contact with much of the perimeter of the brainstem, and six had tumors that extended to the cerebellopontine angle with considerable cervical medullary involvement (below C2).

**Figure 1:**
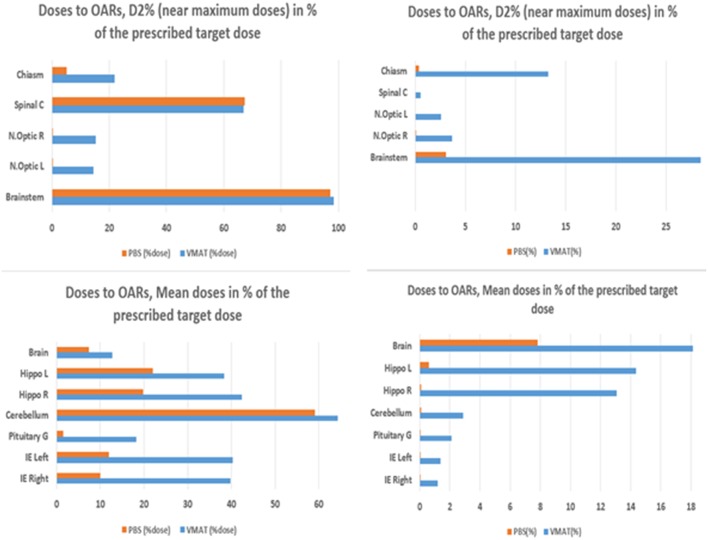
Doses to OARs and near-maximum D2% (top) and mean doses (bottom) as a % of the prescribed target dose. Infratentorial tumor (left panel) and supratentorial tumor (right panel).

The results are summarized in Supplementary Tables 3–8, Figure 1, and Supplementary Figures 2, 3.

### Planning Target Volumes

#### PTV_67.6_

In the case of infratentorial tumor (Supplementary Table 3), IMPT yielded better results for HI (median: 0.06 vs. 0.07, *p*_adj_ < 0.0001, median Δ = −0.015), CI (median: 1.37 vs. 1.51, *p*_adj_ ≤ 0.0001, median Δ = −0.17), and DSC (median: 0.84 vs. 0.78, *p*_adj_ ≤ 0.0001, median Δ = 0.05) than VMAT did. In the case of supratentorial tumor, IMPT also yielded better results for HI (median: 0.05 vs. 0.06, *p*_adj_ = 0.40014, median Δ = −0.006), CI (median: 1.22 vs. 1.32, *p*_adj_ < 0.0001, median Δ = −0.12), and DSC (median: 0.90 vs. 0.85, *p*_adj_ < 0.0001, median Δ = 0.052) than VMAT did (Supplementary Table 6).

#### PTV_59.4_

In the case of infratentorial tumor (Supplementary Table 4), IMPT yielded better results for HI (median: 0.18 vs. 0.20, *p*_adj_ < 0.0001, median Δ = −0.10), CI (median: 1.13 vs. 1.17, *p*_adj_ < 0.0001, median Δ = −0.040), and DSC (median: 0.93 vs. 0.91, *p*_adj_ < 0.0001, median Δ = 0.020) than VMAT did. In the case of supratentorial tumor (Supplementary Table 7), IMPT yielded better results for CO (median: 0.95 vs. 0.93, *p*_adj_ = 0.0088, median Δ = 0.016).

### Organ-at-Risk Volumes

Results are provided for the whole cohort (Supplementary Tables 5, 8). Mean doses are reported for organs with a mainly parallel architecture, while near-maximum (D2%) doses are provided for serial-type organs. Dosimetric results are compared for each OAR. IMPT doses were generally lower than VMAT doses for six OARs (brain, hippocampus, inner ears, pituitary gland, cerebellum, and body), *p*_adj_ < 0.05, for both supra- and infratentorial tumors.

Near-maximum (D2%) doses to the optic nerves, chiasm, and brainstem were significantly lower for IMPT (*p*_adj_ < 0.0001), for both supra- and infratentorial tumors. The whole-body dose was lower for protons (*p*_adj_ < 0.0001).

### Brainstem

Dmean and D2% doses were significantly lower for IMPT than for VMAT (*p*_adj_ < 0.0001), for both supra- and infratentorial tumors. D50% did not differ between IMPT and VMAT in the case of infratentorial tumors, but V_59Gy_ for IMPT was significantly lower in the case of infratentorial tumors (*p*_adj_ < 0.0001, median Δ = −2.465).

### Worst Cases

The worst cases were infratentorial tumors arising from or centered on the lateral recess of the fourth ventricle (i.e., foramen of Luschka). It was more difficult to optimize treatment planning for this tumor location, which we observed in 48.33% of our infratentorial cohort, than for centrally located tumors.

### Comparison With Actually Delivered OAR Doses

**Figure 2:**
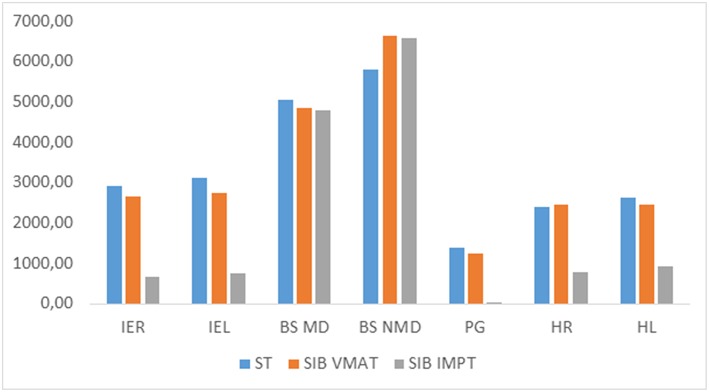
Median of mean and near-maximum doses (cGy) to OARs received at treatment (tt) with SIB-VMAT or SIB-PBS in the case of infratentorial tumor. IER, right inner ear; IEL, left inner ear; BS MD, brainstem mean dose; BS NMD, brainstem near-maximum dose; PG, pituitary gland; HR, right hippocampus; HL, left hippocampus; ST, standard treatment.

**Figure 3:**
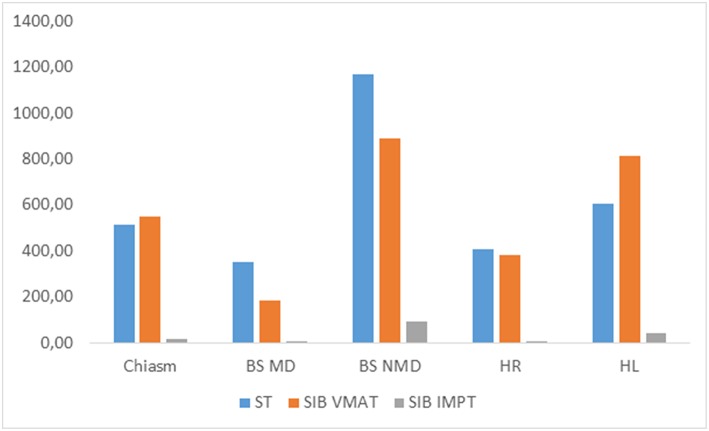
Median of mean and near-maximum doses (cGy) to OARs received at treatment (tt) with SIB-VMAT or SIB-PBS in the case of supratentorial tumor. BS MD, brainstem mean dose; BS NMD, brainstem near-maximum dose; PG, pituitary gland; HR, right hippocampus; HL, left hippocampus; ST, standard treatment.

The additional comparison revealed that, in all patients with infratentorial tumors (Figure 2), mean and near-maximum doses to some OARs in SIB-IMPT were significantly lower than the doses received during the initial treatment. For the brainstem, the mean dose was significantly lower, but the near-maximum dose was significantly higher. In the case of SIB-VMAT, this difference was not significant for any of the OARs except the brainstem where, despite the mean dose being significantly lower, the near-maximum dose was significantly higher. For supratentorial tumors (Figure 3), the mean and near-maximum doses to some OARs were significantly lower with SIB-IMPT, but the difference was not significant with SIB-VMAT.

## Discussion

In the present study, we assessed the feasibility of escalating the radiation dose and quantified the differences between IMPT and VMAT in a large number of patients with pediatric supra- or infratentorial ependymoma. We did this to identify the types of patients who would gain the most from being treated with protons in the case of an escalating dose.

Results showed that in the majority of cases, the target coverage could be retained with a significant reduction in the dose to healthy surrounding tissues. Doses to the brain, hippocampus, inner ears, brainstem, pituitary gland, temporal lobes, cerebellum, chiasm, and optic nerves were significantly lower with IMPT than with VMAT, for both supra- and infratentorial tumors. Doses to the spinal cord of some patients with infratentorial tumor were not significantly lower with IMPT than with VMAT. Indeed, most of the difficult cases for target coverage were infratentorial tumors arising from or centered on the lateral recess of the fourth ventricle (i.e., foramen of Luschka)-a tumor location that is often associated with a poorer prognosis than centrally located tumors (20–23).

We compared VMAT, which is still the most common technique for pediatric radiation therapy, with IMPT. There is no doubt that protons can spare healthy tissue more than VMAT can, and for most of the patients in our cohort, we found considerable sparing of OARs and normal tissue. For the individual patient, this may lead to fewer acute side effects resulting from the radiation treatment, as described recently by Rombi et al. in their overview of clinical results (24).

Furthermore, despite escalating the dose to 67.6 Gy, mean and near-maximum doses to OARs were significantly lower with IMPT. With VMAT, they did not differ significantly from those initially delivered at a median dose of 59.4 Gy for most OARs. The exception was the brainstem, where the near-maximum dose was higher-possibly because of this organ's vicinity to the high-dose PTV. Radiation techniques and the doses that were actually delivered in the plans differed across patients. We nevertheless preferred to retain them all, in order to benefit from the statistical power afforded by having a larger sample, and also to demonstrate that substantially higher doses can be delivered to the tumor bed with SIB-VMAT without increasing doses to OARs, while doses to OARs may even decrease with SIB-IMPT. However, these results should be treated with caution, as the dose constraints applied to the delivery of standard-dose treatments varied slightly between treatment centers.

Based on previous results from our team (25), we assumed that high-dose SIB would not increase the dose to OARs. We ran a comparison on a more limited number of cases (i.e., those with contoured OARs available for actually delivered treatments), which yielded the expected results. We postulated that escalating the dose to the tumor bed can improve outcome in pediatric ependymoma and possibly lower the local recurrence rate in the tumor bed, while sparing the surrounding OARs, compared with standard IMRT dose. This postulate can be criticized, insofar as high doses may cause late side effects and toxicity in children, as they are more radiosensitive than adults on account of their ongoing growth and development (26). Nonetheless, we found that SIB dose escalation did not increase the dose to most OARs with SIB-VMAT, and actually decreased it with SIB-IMPT. Moreover, some studies of CNS reirradiation in pediatric patients (26–29) and irradiation of pediatric or adult brain tumors such as skull-base chordoma (30–32) have reported that OARs can tolerate high doses, without reaching high toxicity rates. In Rombi et al. (30), for instance, high radiation doses were used at levels similar to those used in adult patients [i.e., up to 74 Gy(RBE)]. Proton therapy combined with optimum surgery offers realistic chances of a cure, with acceptable risks of side effects.

Fung et al. (31) reported that no brainstem or spinal cord complications were observed in their series, even though doses of up to 64 and 55 Gy(RBE) were delivered to the surface of these two structures. Merchant et al. (33) reported that dose, volume, and recovery of brainstem function were unrelated in a cohort of patients treated with doses up to 59.4 Gy. Brainstem recovery after surgery for infratentorial ependymoma was time-dependent and mediated by a number of factors, including the number of tumor resections, age at time of irradiation, sex, CSF shunting, and tumor volume. There are no specific data on doses and toxicity to brainstem for the application of the prospective AIEOP protocol (18) to pediatric patients with ependymoma treated with high-dose SRT to tumor residue. Indelicato et al. (34) identified age <5 years, posterior fossa tumor location, and specific dosimetric parameters as factors associated with an increased risk of brainstem toxicity for pediatric brain tumors, including ependymomas, following passively scattered proton therapy (PSPT). They recommended lowering doses to the brainstem as much as possible, and established guidelines to allow for the safe delivery of proton radiation (35). However, as discussed by MacDonald (36), there is still much to be learned about the interplay between the long-term impact of proton therapy on normal tissues and an individual's radiation sensitivity, such that specific adjustments to treatment may ultimately be required to maximize the benefit of therapy and minimize the risks.

Until we have more data on brainstem toxicity with new proton techniques, we would advise against using dose escalation for infratentorial tumors encasing the brainstem or with medullary involvement.

We are not suggesting that SIB should substitute second-look surgery in the case of residue, but it should be seriously considered in the case of unresectable residue or FLAIR abnormalities in post-operative imaging (17). Therefore, we think that one promising avenue would be the careful stratification of the population eligible to undergo the dose increase, based on residue presence (18), advanced imaging factors (17, 37), and molecular biology (38).

The concept of integrated boost adopted in our study differs from the ongoing European protocol that recommends stereotactic boost solely in the case of residual disease. Given the high local relapse rate within fields even without residue, we think that this integrated boost technique is promising and seems safe for supratentorial tumors.

Until now, radiation oncologists have almost exclusively used PSPT or conformal proton therapy (11, 12, 34). IMPT is dosimetrically better than PSPT in terms of homogeneous target coverage and OAR sparing (11, 39, 40). However, current IMPT methods have many drawbacks. In particular, compared with IMRT, they are highly vulnerable to uncertainties.

In current proton therapy practice, the RBE of protons relative to photons is simplistically assumed to have a constant value of 1.1 in all situations. However, it is becoming increasingly obvious that RBE varies widely along the path of a proton beam. As a consequence, the biologically effective dose distributions that are currently delivered may lead to suboptimal treatments and unforeseen local failures or toxicities. Clinical evidence of the variable biological effectiveness of protons in pediatric patients treated for ependymoma was reported in a recent study (41).

Studies investigating the impact of a variable RBE have also reported substantial differences, compared with the use of a constant factor of 1.1 (42–46). At present, RBE-based treatment is not recommended, but LET-guided optimization (47) should improve outcomes.

The first potential limitation of the present study is our use of the geometry-based PTV for IMPT. However, as we wanted to compare the two different modalities, it made more sense to use it for IMPT and VMAT planning. Furthermore, the dose-volume constraints in the pediatric treatment protocol were specifically set for photons, and not for protons. Nonetheless, as there is not yet any consensus in the medical community, PTV-based planning remains the standard of care for proton therapy in France. The second potential limitation is that we did not include a comparison of normal tissue complication probabilities for selected organs and expected tumor control probability, as has been done in some studies (48, 49). However, image-guidance technology may vary for proton and photon radiotherapy, affecting the PTV margins, and therefore, normal tissue complication probabilities, with an attendant impact on the selection of patients eligible for proton therapy (50).

In our study, doses for IMPT were calculated with the pencil beam algorithm. Using Monte Carlo (MC) techniques, or their accelerated variants (accelerated MC), would help to overcome the limitations of such approximations and assumptions. MC methods are also essential for calculating other physical quantities (e.g., LET) required for computing RBE. In our study, however, we used a version of RayStation in which proton MC was not yet available.

Owing to the use of a range shifter, the penumbra was relatively large. This factor needs to be taken into account when looking for ways of improving results. We intuitively assume that the smaller the spot size, the sharper the penumbra and the more conformal the PT dose distribution. Aside from the use of smaller spot sizes, there is now a method for significantly improving the penumbra, by introducing an aperture in the IMPT plan. This technique is now supported in RayStation and is in clinical use in several proton sites. All current systems use the smallest possible spot size for all spots and energies. As reported in Mohan et al. (39), it will be possible to improve IMPT if treatment planning is first improved, by reducing uncertainties in proton range, enhancing adaptive treatment planning and delivery, and considering how to exploit the variability of proton RBE for clinical purposes.

Establishing a radiotherapy treatment plan is a complex procedure, and its quality and results depend on both the planner's expertise and fixed constraints. To allow for unbiased comparisons between IMPT and IMRT for each patient, automation of the treatment planning process should be considered (51) and explored. Then again, these treatment plans are highly personalized, compared with other tumor sites that are more easily automatized (50). Finally, since IMPT will be used largely for pediatric patients, it is important to consider models and measures of the neutron dose contribution induced by the secondary neutrons.

## Conclusions

Dose escalation in pediatric ependymoma with either modulated-intensity photon or proton techniques is feasible in some patients, and in most cases, the use of protons results in a significant reduction in the dose to OARs. Caution needs to be exercised with infratentorial tumors, given the recent data on brainstem toxicity, and dose escalation should be contraindicated in the case of large tumors encasing more than half the brainstem perimeter or with cervical medullary involvement. We think that this approach could be considered in patients with supratentorial tumors with a high risk of relapse (i.e., unresectable residue or post-operative FLAIR abnormalities).

## Ethics Statement

This study was approved by the national French ethics committee and the French data protection authority (CNIL: Commission nationale de l'informatique et des libertés de France).

## Author Contributions

FT contributed to the study design, data collection, data analysis, treatment plan generation, and manuscript writing. AD contributed to data collection, study design, and manuscript revision. LéC performed statistical analysis and manuscript revision. SB, JH, CA, BC-D, VB, LiC, CC, LP, XM, SS, AH, JL, and CK contributed to the clinical aspect of the study design, data collection, and manuscript revision. GH, AlL, FG, and RF contributed to the medical physics aspect of the study design, treatment planning, data collection, and manuscript revision. AnL was the PI of this project, contributed to the study design, validation of treatment planning and manuscript revision, and provided overall guidance.

### Conflict of Interest Statement

The authors declare that the research was conducted in the absence of any commercial or financial relationships that could be construed as a potential conflict of interest.
