# Supplementary material for: Feasibility of Dose Escalation in Patients With Intracranial Pediatric Ependymoma
Source: Front Oncol. 2019 Jun 21;9:531. doi: 10.3389/fonc.2019.00531 (PMC6598548; doi:10.3389/fonc.2019.00531)
Supplement: Supplementary file 1 [file Table_1.DOCX]

**Supplementary Table 1**

Dose‑Volume Constraints for PTVs Used for Optimization

*Note.* D_x%_ = dose received by x% of structure volume; V_xGy_ = percent structure volume of xGy; D_prescr HR_ = prescribed dose to high-risk PTV = 67.6 Gy; D_prescr LR_ = prescribed dose to low-risk PTV =59.4 Gy.

| Structures | Optimization objectives | Optimization priorities |
| --- | --- | --- |
| PTV_67.6 Gy_ | D_50%_ = 67.6 Gy  D_min_ > 100% D_prescr HR_  D_max_ < 102% D_prescr HR_ | Very high  Very high  Very high |
| PTV_59.4 Gy_ – PTV_67.6 Gy_ | D_min_ > 100% D_prescr LR_  D_max_ < 95% D_prescr HR_  D_5%_ < 105% D_prescr LR_ | Very high  High  High |
