# Supplementary material for: Feasibility of Dose Escalation in Patients With Intracranial Pediatric Ependymoma
Source: Front Oncol. 2019 Jun 21;9:531. doi: 10.3389/fonc.2019.00531 (PMC6598548; doi:10.3389/fonc.2019.00531)
Supplement: Supplementary file 2 [file Table_2.DOCX]

**Supplementary Table 2**

Criteria for Plan Validation

| Structure | Dose contraint |
| --- | --- |
| PTV_67.6 Gy_ | D_50%_ = 67.6 y  D_95%_ > 95%D_prescr HR_  D_98%_ > 90% D_prescr HR_  D_0%_ > 107% D_prescr HR_ |
| PTV_59.4 Gy_ - PTV_67.6 Gy_ | D_95%_ > 95% _Dprescr LR_  D_98%_ > 90% _Dprescr LR_ |
| Chiasm | D_max_ < 54 Gy |
| Brainstem | D_mean_ < 54 Gy  D_max_ < 6422 Gy  1‑10 cc < 59 Gy |
| Pituitary gland | D_mean_ < 16 Gy |
| Inner ear R/L | D_max_ < 50 Gy  D_mean_ < 35 Gy |
| Cochlea R/L | D_max_ < 50 Gy  D_mean_ < 35 Gy |
| Eye R/L | D_mean_ < 40 Gy |
| Lens R/L | D_max_ < 10 Gy |
| Optic nerves | D_max_ < 50 Gy |
| Temporal lobes | D_max_ < 60 Gy |
| Spinal cord | D_max_ < 54 Gy |
| Brain PTV | D_mean_ < 20 Gy |
