# Supplementary material for: Feasibility of Dose Escalation in Patients With Intracranial Pediatric Ependymoma
Source: Front Oncol. 2019 Jun 21;9:531. doi: 10.3389/fonc.2019.00531 (PMC6598548; doi:10.3389/fonc.2019.00531)
Supplement: Supplementary file 3 [file Table_3.DOCX]

***Supplementary Table 3:*** Median (Range) Dosimetric Results for Planning Target Volumes (PTV67.6 in the Case of Infratentorial Tumour)

| (n= 60) VMAT IMPT p adjust | Δ(IMPT – VMAT) |
| --- | --- |
| D2% (Gy) **p < 0.0001**  Median 69.983 68.782  (Range) (68.570:71.639) (68.080:70.022)  D50% (Gy) p = 0.1550  Median 67.600 67.600  (Range) (67.600:67.600) (67.600:67.600)  D98% (Gy) p = 0.4404  Median 64.848 64.781  (Range) (62.446:66.120) (63.096:66.120)  HI **p < 0.0001**  Median 0.073 0.057  (Range) ( 0.042: 0.135) ( 0.029: 0.100)  CI **p < 0.0001**  Median 1.510 1.365  (Range) ( 1.140: 2.400) ( 1.090: 1.835)  CO p = 0.1486  Median 0.955 0.960  (Range) ( 0.553: 1.001) ( 0.699: 0.999)  DSC **p < 0.0001**  Median 0.778 0.837  (Range) ( 0.567: 0.906) ( 0.695: 0.930)  Target coverage p = 0.8113  Median 99.514 99.671  (Range) (91.799:100.00) (93.788:100.00) | PTV 67.6: D2% (Gy)  Median -1.069  (Range) (-2.766: 0.277)  PTV 67.6: D50% (Gy)  Median 0.000  (Range) (-0.000: 0.000)  PTV 67.6: D98% (Gy)  Median -0.044  (Range) (-1.309: 1.139)  PTV 67.6: HI  Median -0.015  (Range) (-0.054: 0.021)  PTV 67.6: CI  Median -0.170  (Range) (-0.710: 0.080)  PTV 67.6: CO  Median 0.005  (Range) (-0.054: 0.146)  PTV 67.6: DSC  Median 0.052  (Range) (-0.019: 0.241)  PTV 67.6: Target coverage Median 0.000  (Range) (-1.858: 4.561) |
