# Supplementary material for: Feasibility of Dose Escalation in Patients With Intracranial Pediatric Ependymoma
Source: Front Oncol. 2019 Jun 21;9:531. doi: 10.3389/fonc.2019.00531 (PMC6598548; doi:10.3389/fonc.2019.00531)
Supplement: Supplementary file 4 [file Table_4.DOCX]

***Supplementary Table 4:*** Median (Range) Dosimetric Results for Planning Target Volumes (PTV59.4 in the Case of Infratentorial Tumour)

| (n= 60) VMAT IMPT p adjust | Δ(IMPT – VMAT) |
| --- | --- |
| D2% (Gy) **p < 0.0001**  Median 69.431 68.576  (Range) (68.390:71.639) (66.309:69.703)  D50% (Gy) **p < 0.0001**  Median 65.303 64.698  (Range) (63.171:66.742) (61.294:66.656)  D98% (Gy) p = 0.6196  Median 57.029 57.161  (Range) (51.306:59.843) (53.604:58.327)  HI **p < 0.0001**  Median 0.195 0.179  (Range) ( 0.133: 0.230) ( 0.153: 0.212)  CI **p < 0.0001**  Median 1.170 1.130  (Range) ( 1.040: 1.960) ( 1.000: 1.540)  CO **p = 0.0007**  Median 0.849 0.878  (Range) ( 0.586: 0.955) ( 0.491: 0.965)  DSC **p < 0.0001**  Median 0.913 0.933  (Range) ( 0.655: 0.963) ( 0.784: 0.973)  Target coverage p = 0.1550  Median 99.180 99.544  (Range) (96.916:100.000) (95.369:100.000) | PTV 59.4: D2% (Gy)  Median -0.702  (Range) (-3.712: 0.236)  PTV 59.4: D50% (Gy)  Median -0.646  (Range) (-2.969: 0.154)  PTV 59.4: D98% (Gy)  Median -0.051  (Range) (-2.662: 2.298)  PTV 59.4: HI  Median -0.010  (Range) (-0.053: 0.027)  PTV 59.4: CI  Median -0.040  (Range) (-0.670: 0.130)  PTV 59.4: CO  Median 0.015  (Range) (-0.112: 0.139)  PTV 59.4: DSC  Median 0.020  (Range) (-0.051: 0.183)  PTV 59.4: Target coverage Median 0.017  (Range) (-1.578: 1.529) |
