# Supplementary material for: Feasibility of Dose Escalation in Patients With Intracranial Pediatric Ependymoma
Source: Front Oncol. 2019 Jun 21;9:531. doi: 10.3389/fonc.2019.00531 (PMC6598548; doi:10.3389/fonc.2019.00531)
Supplement: Supplementary file 5 [file Table_5.DOCX]

***Supplementary Table 5****:* Median (Range) Dosimetric Results for Organs at Risk and Healthy Tissue in the Case of Infratentorial Tumour

| (n= 60) VMAT IMPT p adjust | Δ(IMPT – VMAT) |
| --- | --- |
| Brain PTV: Dmean (Gy) **p < 0.0001**  Median (Range) 8.560 ( 3.180:25.187) 4.911( 1.956:17.647)  Brainstem: D2% (Gy) **p < 0.0001**  Median (Range) 66.509(59.430:68.144) 65.756 (57.940:66.995)  Brainstem: D50% (Gy) p = 0.6012  Median (Range) 54.535 (17.540:61.661) 54.195( 6.270:61.569)  Brainstem: Dmean (Gy) **p < 0.0001**  Median (Range) 48.527 (18.390:53.673) 47.973( 6.650:53.801)  Brainstem: Vol 59Gy **p < 0.0001**  Median (Range) 38.060( 2.230:57.310) 35.975( 1.430:61.250)  Inner ear R: Dmean (Gy) **p < 0.0001**  Median (Range) 26.912 (14.050:50.716) 6.798 ( 0.410:45.373)  Inner ear L: Dmean (Gy) **p < 0.0001**  Median (Range) 27.207 (10.740:60.553) 7.913( 0.050:56.380)  Optic nerve R: D2% (Gy) **p < 0.0001**  Median (Range) 10.358( 3.936:33.977) 0.031(0.000:17.160)  Optic nerve L: D2% (Gy) **p < 0.0001**  Median (Range) 9.743 ( 6.360:23.206) 0.033(0.000:19.166)  Chiasm: D2% (Gy) **p < 0.0001**  Median (Range) 13.720( 3.965:36.860) 0.924(0.000:42.988)  Pituitary gland: Dmean (Gy) **p < 0.0001**  Median (Range) 12.355 ( 7.338:16.440) 0.434(0.000:15.882)  Temp lobe R: D2% (Gy) **p < 0.0001**  Median (Range) 43.540( 7.492:68.353) 39.262(0.352:68.746)  Temp lobe L: D2% (Gy) **p < 0.0001**  Median (Range) 41.209( 6.352:69.284) 38.532 (0.160:68.112) | Brain PTV: Dmean (Gy)  Median (Range): -3.357(-7.540:-1.100)  Brainstem: D2% (Gy)  Median (Range): -0.804 (-7.223: 1.177)  Brainstem: D50% (Gy)  Median (Range):-0.172(-11.270: 4.965)  Brainstem: Dmean (Gy)  Median(Range): -0.499(-43.235: 1.201)  Brainstem: Vol 59Gy  Median(Range): -2.465(-20.430: 4.950)  Inner ear R: Dmean (Gy)  Median(Range):-17.810(-27.057:5.961)  Inner ear L: Dmean (Gy)  Median(Range):-18.111(-27.463:4.852)  Optic nerve R: D2% (Gy)  Median(Range):-9.991(-26.395: -0.143)  Optic nerve L: D2% (Gy)  Median(Range):-9.308 (-19.012:-0.501)  Chiasm: D2% (Gy)  Median(Range):-11.59 (-22.183: 6.128)  Pituitary gland: Dmean (Gy)  Median(Range):-10.807(-15.905: 4.569)  Temp lobe R: D2% (Gy)  Median(Range):-3.887 (-29.529: 2.972)  Temp lobe L: D2% (Gy)  Median(Range):-3.691(-19.177: 6.654) |
| Cerebellum: Dmean (Gy) **p < 0.0001**  Median (Range) 43.565(20.612:62.108) 40.124(17.709:61.092)  Hippocampus R: Dmean (Gy) **p < 0.0001**  Median (Range) 28.671(2.035:58.648) 13.352(0.081:58.446)  Hippocampus L: Dmean (Gy) **p < 0.0001**  Median (Range) 25.934(1.755:51.861) 14.913(0.060:48.750)  Spinal cord: D2% (Gy) **p = 0.0050**  Median (Range) 45.207( 1.040:53.887) 45.502( 0.730:53.796)  Body: Dmean (Gy) **p < 0.0001**  Median (Range) 7.208 (2.540:15.815) 4.243( 1.380:11.006) | Cerebellum: Dmean (Gy)  Median (Range):-3.637(-15.075: 0.592)  Hippocampus R: Dmean (Gy)  Median(Range): -12.432(-24.332: 6.862)  Hippocampus L: Dmean (Gy)  Median(Range):-11.616(-22.664:12.043)  Spinal cord: D2% (Gy)  Median(Range): 0.649 (-6.276:31.707)  Body: Dmean (Gy)  Median(Range): -2.959 (-8.279:-1.160) |
