# Supplementary material for: Feasibility of Dose Escalation in Patients With Intracranial Pediatric Ependymoma
Source: Front Oncol. 2019 Jun 21;9:531. doi: 10.3389/fonc.2019.00531 (PMC6598548; doi:10.3389/fonc.2019.00531)
Supplement: Supplementary file 6 [file Table_6.DOCX]

***Supplementary Table 6****:* Median (Range) Dosimetric Results for Planning Target Volumes (PTV67.6) in the Case of Supratentorial Tumour

| (n= 31) VMAT IMPT p adjust | Δ(IMPT – VMAT) |
| --- | --- |
| D2% (Gy) **p < 0.0001**  Median 69.339 68.703  (Range) (68.207:71.546) (68.135:69.326)  D50% (Gy) p = 0.3795  Median 67.600 67.600  (Range) (67.600:67.600) (67.600:67.600)  D98% (Gy) p = 0.2390  Median 65.249 65.295  (Range) (63.668:66.482) (64.227:65.972)  HI **p = 0.0014**  Median 0.060 0.053  (Range) (0.037: 0.101) (0.033: 0.073)  CI **p < 0.0001**  Median 1.320 1.220  (Range) (1.160: 1.750) (1.020: 1.460)  CO p = 0.4172  Median 0.975 0.973  (Range) (0.907: 0.997) (0.930: 0.997)  DSC **p < 0.0001**  Median 0.851 0.896  (Range) ( 0.724: 0.915) ( 0.812: 0.968)  Target coverage p = 0.1428  Median 99.960 99.994  (Range) (96.994:100.000) (98.179:100.000) | PTV 67.6: D2% (Gy)  Median -0.747  (Range) (-3.081: 0.050)  PTV 67.6: D50% (Gy)  Median 0.000  (Range) (-0.000: 0.000)  PTV 67.6: D98% (Gy)  Median -0.150  (Range) (-1.550: 0.930)  PTV 67.6: HI  Median -0.006  (Range) (-0.044: 0.008)  PTV 67.6: CI  Median -0.120  (Range) (-0.290: 0.030)  PTV 67.6: CO  Median 0.003  (Range) (-0.027: 0.073)  PTV 67.6: DSC  Median 0.052  (Range) (-0.010: 0.088)  PTV 67.6: Target coverage  Median 0.012  (Range) (-0.738: 2.921) |
