# Supplementary material for: Feasibility of Dose Escalation in Patients With Intracranial Pediatric Ependymoma
Source: Front Oncol. 2019 Jun 21;9:531. doi: 10.3389/fonc.2019.00531 (PMC6598548; doi:10.3389/fonc.2019.00531)
Supplement: Supplementary file 7 [file Table_7.DOCX]

***Supplementary Table 7****:* Median (Range) Dosimetric Results for Planning Target Volumes (PTV59.4) in the Case of Supratentorial Tumour

| (n= 31) VMAT IMPT p adjust | Δ(IMPT – VMAT) |
| --- | --- |
| D2% (Gy) **p** **< 0.0001**  Median 69.126 68.583  (Range) (66.122:70.949) (68.060:69.133)  D50% (Gy) **p** **< 0.0001**  Median 66.120 65.808  (Range) (64.361:67.266) (64.161:67.200)  D98% (Gy) p = 0.3505  Median 58.433 58.195  (Range) (55.670:60.500) (56.707:59.790)  HI p = 0.1472  Median 0.160 0.155  (Range) (0.125: 0.211) (0.126: 0.192)  CI p = 0.2390  Median 1.150 1.160  (Range) (1.030: 1.442) (1.020: 1.520)  CO **p = 0.0088**  Median 0.933 0.945  (Range) (0.753: 0.969) (0.791: 0.994)  DSC p = 0.1627  Median 0.918 0.921  (Range) ( 0.817: 0.979) (0.790: 0.985)  Target coverage p = 0.4172  Median 99.921 99.891  (Range) (98.340:100.000) (98.704:100.000) | PTV 59.4: D2% (Gy)  Median -0.552  (Range) (-2.662: 2.453)  PTV 59.4: D50% (Gy)  Median -0.254  (Range) (-1.195: 0.206)  PTV 59.4: D98% (Gy)  Median -0.078  (Range) (-1.409: 1.670)  PTV 59.4: HI  Median -0.003  (Range) (-0.040: 0.018)  PTV 59.4: CI  Median -0.020  (Range) (-0.240: 0.200)  PTV 59.4: CO  Median 0.016  (Range) (-0.080: 0.150)  PTV 59.4: DSC  Median 0.008  (Range) (-0.070: 0.094)  PTV 59.4: Target coverage  Median 0.017  (Range) (-0.405: 0.900) |
