# Supplementary material for: Feasibility of Dose Escalation in Patients With Intracranial Pediatric Ependymoma
Source: Front Oncol. 2019 Jun 21;9:531. doi: 10.3389/fonc.2019.00531 (PMC6598548; doi:10.3389/fonc.2019.00531)
Supplement: Supplementary file 8 [file Table_8.DOCX]

***Supplementary Table 8****:* Median (Range) Dosimetric Results for Organs at Risk and Healthy Tissue in the Case of Supratentorial Tumour.

| (n= 31) VMAT IMPT p adjust | Δ (IMPT – VMAT) |
| --- | --- |
| Brain PTV: Dmean (Gy) **p < 0.0001**  Median(Range) 12.251(4.986:20.391) 5.286(1.802: 9.671)  Brainstem: D2% (Gy) **p < 0.0001**  Median(Range) 19.250 (0.359:63.135) 2.064(0.000:62.998)  Brainstem: D50% (Gy) **p < 0.0001**  Median(Range) 2.130(0.196:41.553) 0.005(0.000:14.314)  Brainstem: Dmean (Gy) **p < 0.0001**  Median(Range) 4.273(0.182:34.351) 0.168(0.000:23.220)  Brainstem: Vol 59Gy p = 0.1441  Median(Range) 0.000(0.000:28.400) 0.000(0.000:18.720)  Inner ear R: Dmean (Gy) **p < 0.0001**  Median(Range) 1.550 (0.000:15.264) 0.000(0.000:16.489)  Inner ear L: Dmean (Gy) **p = 0.0007**  Median(Range) 0.930 ( 0.015:55.853) 0.000 (0.000:51.901)  Optic nerve R: D2% (Gy) **p < 0.0001**  Median(Range) 2.473(0.000:28.159) 0.001(0.000:31.999)  Optic nerve L: D2% (Gy) **p < 0.0001**  Median(Range) 1.758(0.000:28.840) 0.000(0.000:27.207)  Chiasm: D2% (Gy) **p < 0.0001**  Median(Range) 8.96(0.083:51.081) 0.257(0.000:48.305)  Pituitary gland: Dmean (Gy) **p < 0.0001**  Median(Range) 1.437(0.079:15.683) 0.007(0.000:15.910)  Temp lobe R: D2% (Gy) **p = 0.0001**  Median(Range) 15.925(0.359:69.578) 0.165(0.000:68.856)  Temp lobe L: D2% (Gy) **p = 0.0003**  Median(Range) 24.236(0.272:70.110) 2.174 ( 0.000:68.692) | Brain PTV: Dmean (Gy)  Median(Range):-7.741 (-13.616:-3.184)  Brainstem: D2% (Gy)  Median(Range):-1.675(-29.402: 7.607)  Brainstem: D50% (Gy)  Median(Range):-1.634 (-30.684:-0.196)  Brainstem: Dmean (Gy)  Median(Range):-3.300(-26.592:-0.182)  Brainstem: Vol 59Gy  Median(Range): 0.000(-9.680: 0.050)  Inner ear R: Dmean (Gy)  Median(Range):-0.792(-14.999: 1.226)  Inner ear L: Dmean (Gy)  Median(Range):-0.480 (-15.099: 5.756)  Optic nerve R: D2% (Gy)  Median(Range)-1.001(-19.169: 3.840)  Optic nerve L: D2% (Gy)  Median(Range):-0.877(-16.930: 2.502)  Chiasm: D2% (Gy)  Median(Range):-1.775(-24.160: 4.577)  Pituitary gland: Dmean (Gy)  Median(Range):-0.831(-15.646: 4.294)  Temp lobe R: D2% (Gy)  Median(Range):-1.733(-31.430: 9.906)  Temp lobe L: D2% (Gy)  Median(Range):-1.419(-24.294:20.952) |
| Cerebellum: Dmean (Gy) p < 0.0001  Median(Range) 1.945(0.125:29.530) 0.072(0.000:23.560)  Hippocampus R: Dmean (Gy) p < 0.0001  Median(Range) 8.842(0.340:53.652) 0.070 ( 0.000:53.311)  Hippocampus L: Dmean (Gy) p < 0.0001  Median(Range) 9.700(0.362:67.705) 0.420(0.000:67.591)  Spinal cord: D2% (Gy) p < 0.0001  Median(Range) 0.363(0.000: 9.297) 0.000(0.000: 0.150)  Body: Dmean (Gy) p < 0.0001  Median(Range) 7.174( 2.216:15.630) 3.893 (1.465:10.310) | Cerebellum: Dmean (Gy)  Median(Range):-0.870 (-20.013: 0.780)  Hippocampus R: Dmean (Gy)  Median(Range):-2.650(-34.971: 0.770)  Hippocampus L: Dmean (Gy)  Median(Range):-3.017(-27.093: 1.236)  Spinal cord: D2% (Gy)  Median(Range):-0.363(-9.222: 0.000)  Body: Dmean (Gy)  Median(Range):-3.198 (-8.909:-0.751) |
