# Supplementary material for: Feasibility of Dose Escalation in Patients With Intracranial Pediatric Ependymoma
Source: Front Oncol. 2019 Jun 21;9:531. doi: 10.3389/fonc.2019.00531 (PMC6598548; doi:10.3389/fonc.2019.00531)

**Supplemenatry materials**

Supplementary Fig.1


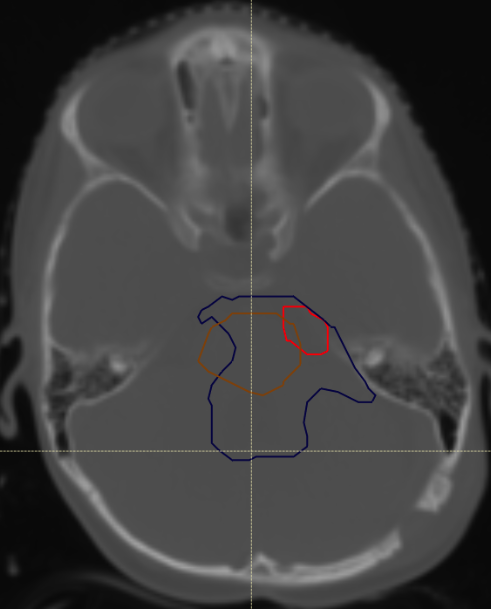

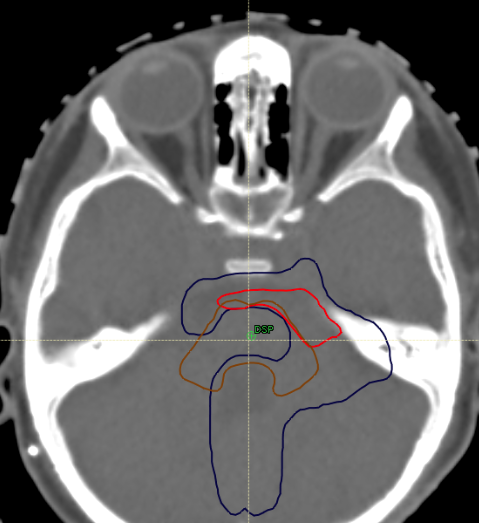

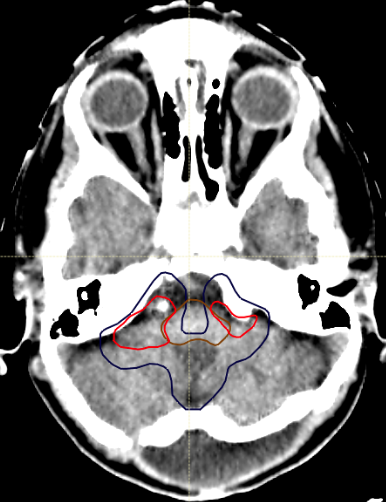


*Three examples of patients who were not replanned and were not eligible for dose escalation, as their tumour was too large to allow a boost to be delivered in the vicinity of the brainstem: tumour residue outlined in red, tumor bed in blue, and brainstem in brown.*

**Supplementary Fig.2**

*Dose distribution for one representative patient with VMAT (top) and one with IMPT (bottom). Red outline: PTV_67.6_. This colour-washed figure shows the dose distribution from 25 Gy to 67.6 Gy. Better sparing of some OARs was observed with IMPT than with VMAT.*


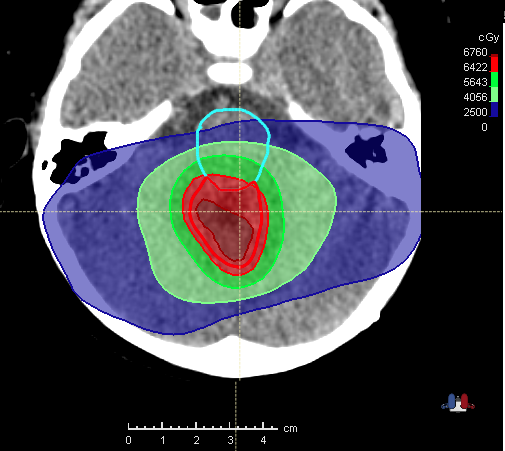

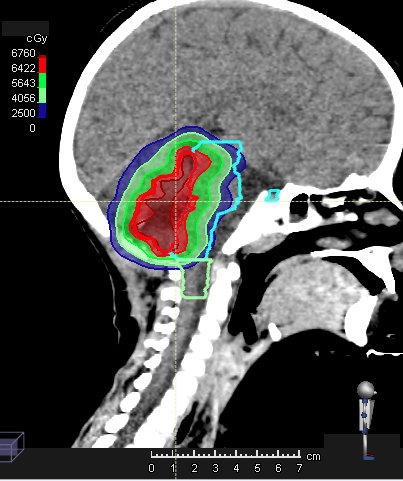

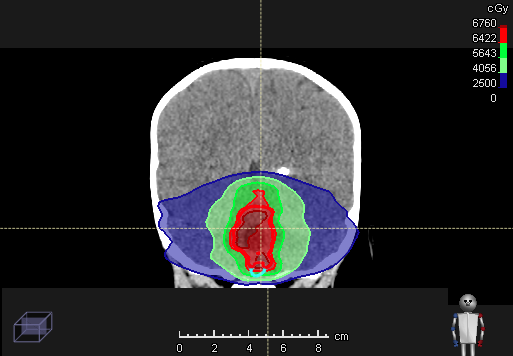


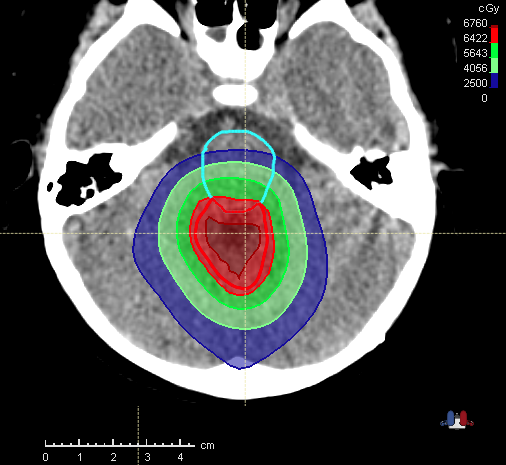

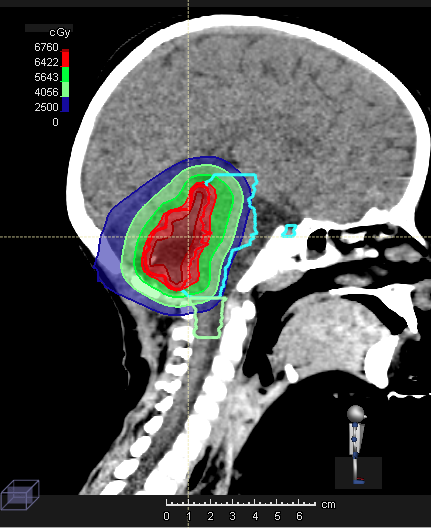

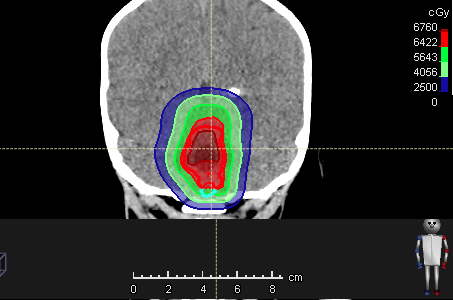


**Supplementary Fig.3**

*Dose‑volume histogram of PTV_67.6 Gy_, PTV_59.4_ Gy, brainstem, right and left inner ear, and spinal cord, for one representative patient with VMAT or IMPT*.


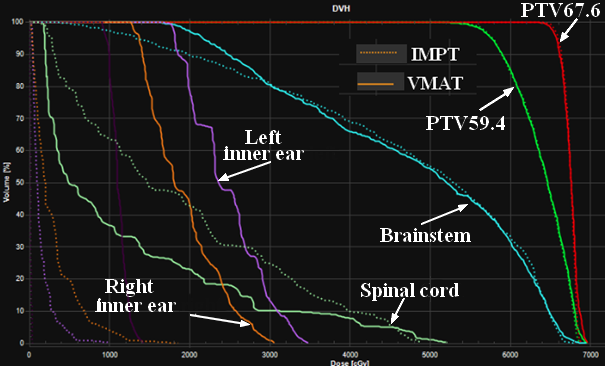

Supplement: Supplementary file 9 [file Data_Sheet_1.docx]
